# Post-treatment work patterns amongst survivors of lymphoma treated with high-dose chemotherapy with autologous stem-cell transplantation

**DOI:** 10.1186/s12885-021-07836-2

**Published:** 2021-02-08

**Authors:** Kjersti Helene Hernæs, Knut B. Smeland, Unn-Merete Fagerli, Cecilie E. Kiserud

**Affiliations:** 1 Norwegian Institute of Public Health, PO Box 222, Skøyen, N-0213 Oslo, Norway; 2 National Advisory Unit for Late Effects after Cancer Treatment, Oslo University Hospital, Radiumhospitalet, PO 4953, Nydalen, N-0424 Oslo, Norway; 3 Department of Oncology, St. Olavs hospital HF, Postboks 3250 Torgarden, 7006 Trondheim, Norway; 4 Institute for Clinical and Molecular Medicine (IKOM), The Norwegian University of Science and Technology (NTNU), Olav kyrres gate 9, 7006 Trondheim, Norway; 5 Previous affiliation: Research Support Services, Oslo University Hospital, Sogn Arena, Klaus Torgårds vei 3, 3. Floor, 0372 Oslo, Norway

**Keywords:** Lymphoma, HDT-ASCT, Late effects, Employment, Work ability, Withdrawal

## Abstract

**Background:**

This study describes post-treatment work patterns in lymphoma survivors treated with high-dose chemotherapy with autologous stem-cell transplantation (HDT-ASCT). It aims to identify determinants for labour force participation and exclusion after HDT-ASCT.

**Methods:**

All survivors treated with HDT-ASCT for lymphoma in Norway between 1995 and 2008, aged ≥18 years at HDT-ASCT and alive at survey in 2012–2013 were eligible. We divide survivors by current employment status (full-time, part-time and unemployed). Main outcomes are current employment status, work hours and work ability. *Withdrawals* are patients employed when diagnosed but not before HDT-ASCT.

**Results:**

Of the 274 who completed the survey, 82% (*N* = 225) were included in the final analyses. Mean age at survey was 52 years, 39% were female, 85% were employed when diagnosed, 77% before HDT-ASCT and 69% at survey. Employment before HDT-ASCT corresponds with a higher probability of employment at survey for a given symptom burden. In the most extensive statistical model, it increases with 37.3 percentage points. Work hours amongst withdrawals plummet after HDT-ASCT while work ability shows a rebound effect. The potential economic gain from their re-enter into the work force equals 70% of the average annual wage in Norway in 2012.

**Conclusions:**

For a given symptom burden, staying employed throughout diagnosis and treatment is associated with a higher probability of future employment. These results favour policies for labour force inclusion past diagnosis and treatment increasing cancer survivors’ probability of future employment. However, we need more research on withdrawal mechanisms, and on policy measures that promote inclusion.

## Background

High-dose chemotherapy with autologous stem-cell transplantation (HDT-ASCT) is a potentially curative treatment option for selected lymphoma patients. It is associated with severe acute and late adverse effects. Potential late adverse effects include secondary cancers, cardiovascular disease, peripheral neuropathies, hormonal disturbances, chronic fatigue and mental distress [1], causing physical and mental strain, and challenging patients’ work ability. Due to higher treatment-related mortality and morbidity with increasing age, HDT-ASCT is usually reserved for patients < 65–70 years [2], and most lymphoma patients treated with HDT-ASCT are therefore within working age, with potentially numerous years left until retirement. Maintaining work ability and employment is thus an important issue not only for the individual survivor after HDT-ASCT but also for society as a whole. In a previous study, our group studied employment patterns and associated factors for this patient group [3]. We found psychosocial factors to be associated with labour market withdrawal at follow-up, but hardly any lymphoma-related variables. An extensive body of research relates to absenteeism in work life [4, 5], lending support to the hypothesis that (sickness) absence leads to more absence.

In this article, we describe work-related outcomes amongst lymphoma survivors treated with HDT-ASCT. We have access to data describing work life parameters for lymphoma survivors treated with HDT-ASCT before onset of illness, during and after treatment. From this material, we also study post-treatment work patterns and try to identify determinants for stable labour force participation and exclusion post treatment.

The aims of the present study are to:

1. Investigate factors affecting labour force participation in lymphoma survivors after HDT-ASCT.
2. Compare work ability and work hours for withdrawals (patients who were employed when diagnosed but not before HDT-ASCT) and non-withdrawals.
3. Assess the economic loss of income related to withdrawal.

We postulate the following hypothesis using this dataset: Withdrawal from work life has a causal and negative effect on future work participation.

## Methods

### Patients

**Fig. 1:**
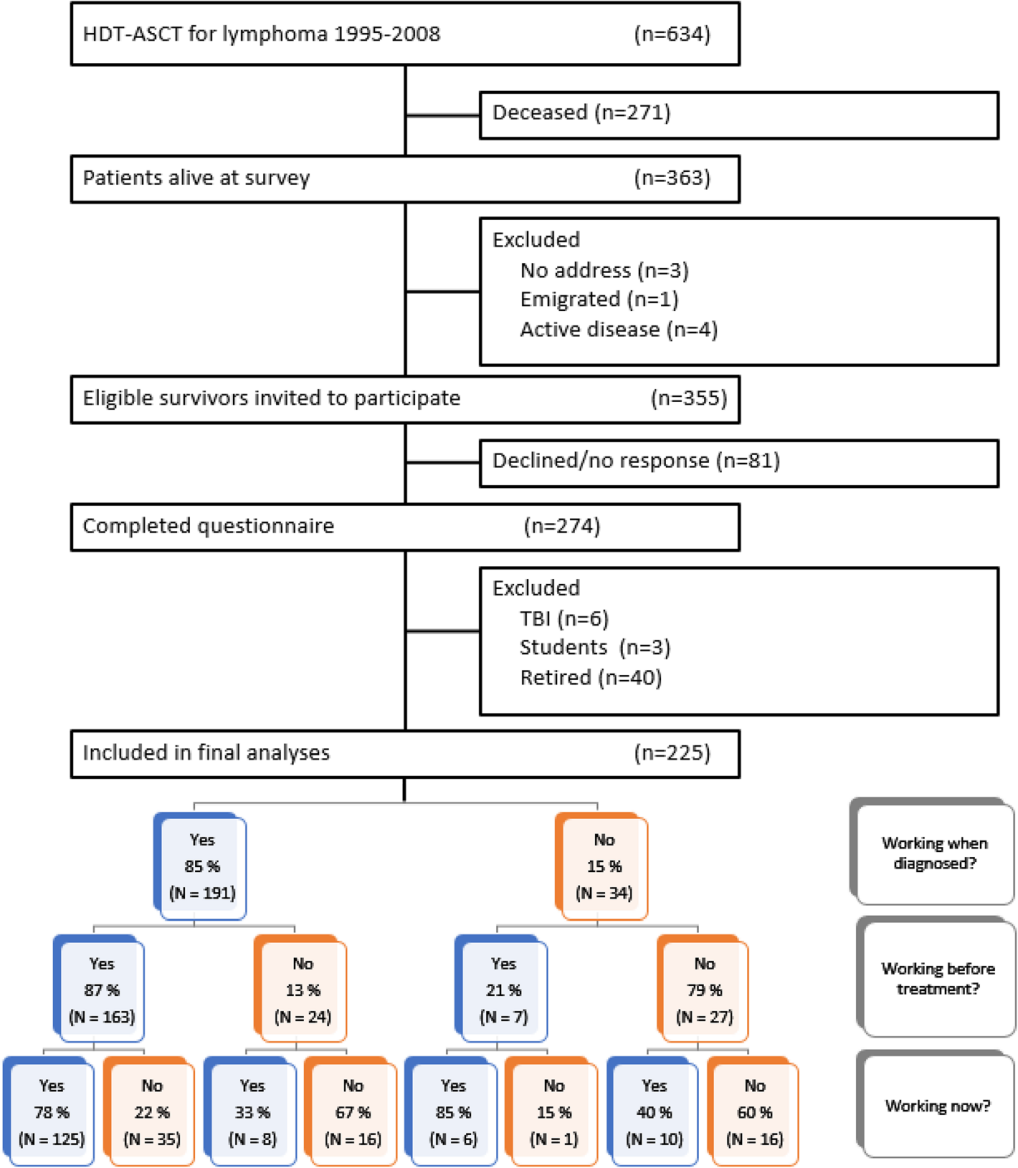
Flowchart and tree showing who works when. Flowchart of the study and tree showing who works when diagnosed, before treatment and at survey (three time points). Withdrawal is defined as moving from a blue box (yes = inclusion) to a red box (no = exclusion). Deviances in sums due to missing values (8 total)

The data source is a national multicentre cross-sectional follow-up study where all survivors treated with HDT-ASCT for lymphoma in Norway between 1995 and 2008, aged ≥18 years at HDT-ASCT, alive at survey, residing in Norway and currently not undergoing systemic therapy for active malignancy were eligible and invited to participate (*n* = 355, Fig. 1). The survey, performed in 2012–2013, consisted of a detailed self-reported questionnaire with a set of well-established patient reported outcome measures [3, 6], a comprehensive out-patient clinical examination [7], as well as data retrieved from patients’ charts and the clinical quality register for lymphoma at Oslo University Hospital (OUH). For the present study, we include only survivors treated with BEAM (carmustine, etoposide, cytarabine and melphalan), which has been the standard high-dose regimen in Norway since 1995, excluding six survivors treated with total body irradiation (TBI). In Norway, all citizens are entitled to old-age pension from 67 years of age. Respondents who reported receiving old-age pension (*n* = 40) or being students (*n* = 3) at survey are excluded in the present study since the focus here is on work patterns before and after treatment, and these two groups are not considered part of the formal labour force. In general, the survey had a low percentage of missing data.

### Treatment

Survivors are categorized according to primary lymphoma entity: Hodgkin lymphoma (HL), indolent non-Hodgkin lymphoma (NHL) and aggressive NHL, number of regimens prior to HDT-ASCT, and whether they experienced relapse after HDT-ASCT or not [3]. Body Mass Index (BMI) is from the clinical examination (kg/m2). We replaced 36 missing values using statistical measures based on self-reported BMI.

### Main outcomes

Patients were asked to retrospectively report their employment status when first diagnosed and before HDT-ASCT, as well as their current work situation (at survey), according to eleven categories. We use this information to construct three categories for employment status; fulltime workers (having a fulltime job, being self-employed, or on sick leave), part-time workers (part-time job), or not employed (unemployment insurance, disability insurance, temporary disability insurance, or homemaker).

Patients rated their work ability on a scale of 1 to 10 (where 10 is best), both as they perceived it when answering the survey, and how they remembered it before onset of illness (i.e. when first diagnosed). They were also asked to report the number of weekly work hours at survey and when diagnosed. Their *current employment status*, *current work hours* and *current work ability* are the main outcomes in our analyses.

Based on their current work situation (at the time of survey), we construct a three-part categorical variable for employment; full-time workers, part-time workers and not employed (Table 1), and a binary variable for being employed at follow-up (not distinguishing between full and part time) or not.

**Table 1:** Descriptives

Variable description	Total	Full-time workers	Part-time workers	Not employed
N	225	113	40	68
*Current work situation (from questionnaire)*				
Background variables	*Per cent*	*Per cent*		
Female	39	21	63	54
Married (missing n = 1)	74	72	73	75
Higher education (missing n = 1)	47	51	54	37
	*Mean (sd)*	*Mean (sd)*		
Age at diagnosis	40 (12.9)	39 (12.7)	40 (13.3)	42 (12.3)
Age at treatment	43 (12.9)	42 (12.3)	43 (14.0)	45 (12.6)
Age at questionnaire	52 (11.6)	51 (10.7)	52 (13.5)	54 (11.6)
Body Mass Index	26 (4.9)	26 (4.0)	26 (6.0)	26 (5.6)
Time: Diagnose to HDT-ASCT	2.9 (3.8)	2.9 (4.32)	3.1 (3.7)	2.7 (2.8)
Time: HDT-ASCT to survey	8.7 (3.7)	8.7 (3.8)	9.1 (3.7)	8.5 (3.6)
Labour market characteristics	*Per cent*	*Per cent*		
Employed at diagnosis	85	89	90	75
Employed before HDT-ASCT (missing n = 4)	77	91	79	53
Employed at survey (missing n = 4)	69	100	100	0
	*Mean (sd)*	*Mean (sd)*		
Work hours at diagnosis (missing *n* = 32)	34 (13.3)	36 (12.2)	34 (11.0)	31 (14.6)
Work hours if employed now	21 (18.6)	34 (12.6)	21 (12.8)	0 (0)
Work ability at diagnosis (missing *n* = 20)	8.5 (2.8)	9.3 (1.5)	8.9 (2.7)	6.8 (3.9)
Work ability at survey (missing *n* = 26)	6.1 (3.2)	7.9 (2.1)	5.8 (2.0)	2.8 (2.9)
Health-related characteristics	*Per cent*	*Per cent*		
Heart disease (missing n = 1)	9	8	8	12
Second cancer	11	7	10	19
Relapse after HDT-ASCT	21	18	28	24
Chronic fatigue (missing *n* = 1)	33	21	43	49
Anxiety	21	12	18	37
Lymphoma diagnose				
➢ Hodgkin’s lymphoma	27	28	25	26
➢ Aggressive lymphoma	65	65	70	62
➢ Indolent lymphoma	8	7	5	12
Treatment lines before HDT-ASCT				
➢ One	28	33	23	21
➢ Two	59	54	63	65
➢ More than two	14	13	15	15

Sample descriptives with sociodemographic and health variables and work-related characteristics. Deviancies due to missing values (of the 225 included in the final analyses, four had missing values for current work situation and could therefore not be categorised as full time, part time or not working). HDT-ASCT: High-dose chemotherapy with autologous stem-cell transplantation, sd: Standard deviation. Fulltime workers: Fulltime job (94) Self-employed (18) Sickleave (1). Part-time workers: (40). Not employed: unemployment insurance (2), disability insurance (50), temporary disability insurance (15), homemaker (1).

For these questions, we have used a modified version of the Work Ability Index (WAI) [8], and questions developed by our group in previous studies [3, 9].

### Withdrawal from work life

We use the three questions that pin their employment status to three points in time (when diagnosed, before HDT-ASCT, and at survey) to create the tree shown in Fig. 1 to describe withdrawal from work life. We postulate that once you move from a blue box (inclusion) to a red box (exclusion) it is harder to return to a blue one. We refer to this as “withdrawal” from work life, conditioned on inclusion at the time of diagnosis, thus only defined for the left-hand side of the tree. Non-withdrawals are the respondents on the left branch of the left-hand side of the tree (three positives, *n* = 125).

### Pseudo panels for withdrawals and non-withdrawals

We exploit the variation in time from when a patient received HDT-ASCT, until he or she completed the questionnaire. We construct three intervals: 3–7 years, 8–12 years and 13 years or more, of relatively equal size. We thus simulate panel data, creating ‘pseudo panels’ for current work hours and work ability, supplemented with work hours and work ability from when they were first diagnosed.

The term ‘pseudo panel’ refers to the fact that we do not have the opportunity to follow the same patients over time. Instead, we group respondents according to how long it has been from the time they received treatment (HDT-ASCT) until they completed the questionnaire.

### Ratios for work hours and work ability

We calculate ratios for work hours as *work hours in the withdrawal group* divided by *work hours amongst those who stayed employed from diagnosis to survey*, repeating the calculation for work ability.

### Economic loss

We estimate an economic loss from the difference in work hours between the withdrawal group and the non-withdrawals using wage statistics from Statistics Norway (https://www.ssb.no/en/statbank/table/08057). We estimate an expected mean wage for the withdrawal group using monthly earnings (NOK) for 2012, covering all employees, matching them by gender and level of education.

### Explanatory variables

We define marriage as being in a paired relationship, and higher education as more than 12 years of education. We construct two binary variables for somatic illnesses; one for having had one or more of three heart diseases (myocardial infarction, angina pectoris or heart failure), and another for second cancers (new cancer diagnosis, other than lymphoma). Chronic fatigue is assessed according to the Fatigue Questionnaire [10], containing 11 items concerning physical (7 items) and mental (4 items) fatigue during the last month. Two additional items cover duration and extent of fatigue. Responses are dichotomised (0 and 1 scored as 0, and 2 and 3 scored as 1), with CF defined as sum score of ≥4 of the dichotomised responses with duration of ≥6 months. Anxiety is derived from the Hospital Anxiety and Depression Scale (HADS), consisting of an anxiety and a depression subscale with seven items each [11]. Each item is scored from 0 (not present) to 3 (highly present), and anxiety caseness is defined as a sum score of ≥8 on the anxiety subscale.

### Statistical analyses

We run a multinomial logistic regression, allowing for comparison between more than two groups (Table 2). The dependent variable was the categorical three-part variable divided into *not working* (base outcome), *part time (work)* and *full time (work)* at the time of survey. We include six covariates, relaxing the rule of thumb of 10 events per variable in logistic regression [12]. Employment before treatment is our main variable of interest. Gender and age at survey are necessary individual characteristics. We choose second cancers, chronic fatigue, and anxiety based on their significance in the regression models (Table 2) and their clinical relevance. Other covariates were tested, but they had limited explanatory power, and were not included in the proceeding analyses.

**Table 2:** Results from multinomial logistic regression

Variable description	Relative risk ratio	z	*p*-value	[95 % CI]
Not working at survey (base outcome)				
Working part time at survey				
Employed before HDT-ASCT	4.17	2.82	0.005	[1.54 11.27]
Female	2.00	1.52	0.128	[0.82 4.88]
Age at survey	0.98	-1.28	0.200	[0.94 1.01]
Second cancer	0.39	-1.45	0.148	[0.11 1.40]
Chronic fatigue	0.98	-0.04	0.972	[0.40 2.40]
Anxiety	0.24	-2.49	0.013	[0.08 0.74]
Constant	-0.90	-0.10	0.922	[0.10 8.23]
Working full time at survey				
Employed before HDT-ASCT	9.57	4.66	0.000	[3.70 24.78]
Female	0.34	-2.68	0.007	[0.15 0.74]
Age at survey	0.95	-3.02	0.003	[0.92 0.98]
Second cancer	0.31	-2.13	0.033	[0.10 0.91]
Chronic fatigue	0.34	-2.65	0.008	[0.15 0.75]
Anxiety	0.32	-2.44	0.015	[0.12 0.80]
Constant	14.84	2.69	0.007	[2.08 106.07]

Being employed before HDT-ASCT (high-dose chemotherapy with autologous stem-cell transplantation) increases probability of employment at survey, part or full time, compared to not being employed (base outcome). *CI* Confidence Interval

We run five regression models with the binary variable for employment (i.e. employed or not) at follow-up as the dependent variable, conditioned on being employed at diagnosis (Table 3). In model 1, employment before treatment is the sole covariate. We expand the model stepwise, adding new covariates in each step. In model 2, we include the sociodemographic variables gender, relationship status, education and age at survey. In model 3, we add the somatic health variables BMI, heart disease, second cancer and relapse of lymphoma. Next, we add the mental health variables chronic fatigue and anxiety (model 4). In model 5, we add the number of treatment lines before HDT-ASCT and lymphoma type.

**Table 3:** Results from the five regression models

Variable description	Model 1	Model 2	Model 3	Model 4	Model 5
N	184	183	182	182	182
Employed at survey	Yes	Yes	Yes	Yes	Yes
Constant	0.333***	0.701***	0.812**	0.955***	0.962***
	(3.85)	(3.70)	(3.20)	(3.79)	(3.71)
Employed before HDT-ASCT	0.448***	0.445***	0.436***	0.384***	0.373***
	(4.83)	(4.70)	(4.62)	(4.09)	(4.00)
Female		−0.094	−0.066	−0.034	− 0.040
		(−1.43)	(−1.00)	(− 0.52)	(0.60)
Married		−0.125	−0.168*	− 0.146*	−0.143*
		(−1.69)	(−2.31)	(−2.05)	(−2.01)
Higher education		0.040	0.073	0.054	0.072
		(0.63)	(1.20)	(0.90)	(1.18)
Age at survey		−0.005	−0.004	−0.005	−0.004
		(−1.63)	(−1.36)	(−1.82)	(−1.15)
Body Mass Index			−0.005	−0.004	− 0.006
			(−0.71)	(−0.57)	(− 0.91)
Heart disease			0.076	0.095	0.063
			(0.66)	(0.83)	(0.55)
Second cancer			−0.444***	−0.410***	−0.474***
			(−4.34)	(−4.09)	(−4.54)
Relapse			0.104	0.078	0.138
			(1.30)	(0.98)	(1.65)
Chronic fatigue				−0.126*	−0.109
				(−1.93)	(−1.65)
Anxiety				−0.176*	−0.192*
				(−2.20)	(2.40)
Treatment lines					
One					*(base outcome)*
Two					0.009
					(0.13)
More than two					−0.168
					(−1.57)
Lymphoma type					
Hodgkin					*(base outcome)*
Aggressive					−0.008
					(0.09)
Indolent					−0.143
					(−1.12)
Variation in model explained (%)	0.11	0.15	0.23	0.28	0.30

Statistics: Each coefficient shows how the probability of employment at survey relates to the various covariates (* p < 0.05; ** p < 0.01; *** p < 0.001; t-values in parentheses).

Employment before treatment, conditioned on being employed at onset, stays significant throughout all five regression models (* *p* < 0.05; ** *p* < 0.01; *** *p* < 0.001; t-values in parentheses). HDT-ASCT = high-dose chemotherapy with autologous stem-cell transplantation.

We use a standard t-test to test whether the difference in means between withdrawals’ and non-withdrawals’ work hours and work ability at more than 13 years is different from the difference in means at onset. We restrict the test to the patients observed at diagnosis and 13 years or more after treatment. We test the null hypothesis, that there is no difference in means.

We exclude respondents with missing observations from the analyses when the relevant variable enters the equation. Despite a somewhat higher incidence of missing values for work hours and work ability, we choose not to use statistical measures to replace them, considering the risk of manipulating the results too great. We do not have additional information (as we did for BMI), which could be used to estimate missing values, and would therefore have to rely on imputations based on other respondents’ values.

### Ethics

The South-East Regional Committee for Medical and Health Research Ethics (REC South East) approved the study, and all participants gave written informed consent.

## Results

### Attrition analysis

There are no differences between the participants and non-participants with regard to age at diagnosis, HDT-ASCT or survey, nor gender, observation time or lymphoma entity.

### Patient characteristics

In total, 274 survivors completed the questionnaire (77% of eligible survivors), 49 were excluded, leaving 225 respondents for the final analyses (Fig. 1).

In total, 39% of the participants are female, 74% are in a paired relationship, and 47% have higher education. Their mean age was 40, 43 and 52 years at diagnosis, HDT-ASCT, and survey, respectively (Table 1).

Labour market characteristics show that 85% of the participants were employed when diagnosed. They worked on average 34 h per week. On a scale of 1 to 10, their work ability averaged 8.5. At the time of the survey, median 10.8 years later, 69% were employed, they worked 21 h per week, and their work ability was 6.1 (Table 1). The 99% confidence intervals show no overlap for the respective means.

### Factors associated with being employed at survey

Results from the multinomial logistic regression model show that being employed before HDT-ASCT increases the probability of employment at survey, part or full time, compared to not being employed (base outcome) (Table 2). The other covariates; female gender, higher age, diagnosed with second cancers, chronic fatigue and anxiety; reduce the probability of working full time at survey (coefficients < 1), while anxiety reduces the probability of working part time.

### Employment before HDT-ASCT positively correlated with later employment

The main result from the five regression models (Table 3) is that employment before HDT-ASCT is significantly and positively correlated with employment at survey throughout all five models. The constant in model 1 predicts a 33% probability of being employed at survey if *not* employed before HDT-ASCT, whereas being employed before HDT-ASCT adds 44.8 percentage points to this probability. In models two to five, employment before HDT-ASCT adds 44.5, 43.6, 38.4, and 37.3 percentage points, respectively.

Three covariates; second cancers, anxiety, and being in a paired relationship; are associated with a lower probability of employment at follow-up. Results from the most extensive model (5) suggest that getting a second cancer diagnosis reduces the probability of employment at survey by 47.4 percentage points. Anxiety is associated with a reduction in this probability of 19.2 percentage points, while being in a paired relationship is associated with a reduction of 14.3 percentage points. The remaining health variables have no significant effect on the probability of future employment.

### Withdrawal versus non-withdrawal group

**Fig. 2:**
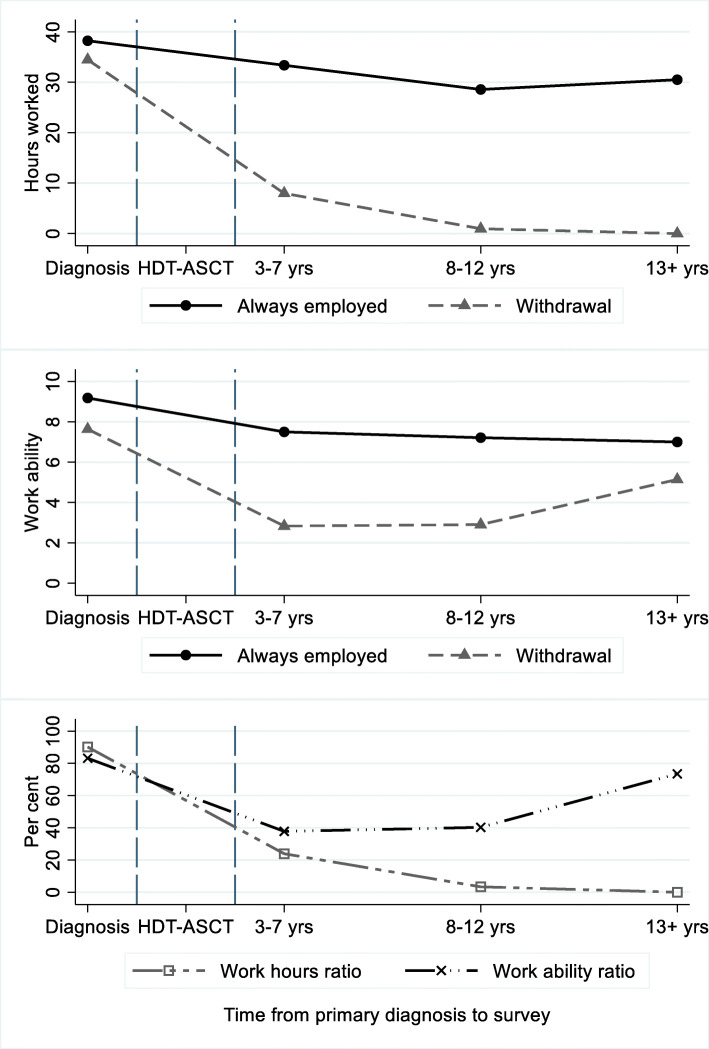
Pseudo panels for work hours and work ability. The two upper panels show pseudo panels for work hours and work ability, for withdrawals and non-withdrawals (always employed). Last panel shows rebound effect for withdrawals’ work ability but not for their work hours. Ratios estimated as withdrawals’ work ability (work hours) as share of non-withdrawals’ work ability (work hours) from diagnose to survey

In Fig. 2 (the top two panels), we show trajectories for the pseudo panels for work hours and work ability for non-withdrawals and withdrawals; at diagnosis, their average reported weekly work hours were 38.2 and 34.5, respectively, similar to a regular workweek (37.5 h). Non-withdrawals rated their work ability at 9.2, while withdrawals rated theirs at 7.6, on average. After HDT-ASCT, average weekly work hours in the withdrawal group drop to 7.1 amongst those who answered the survey 3–7 years later. In the group who responded 8–12 years later, it drops to 1.1, while it is 0 for those responding ≥13 years after HDT-ASCT. The drop in work hours for non-withdrawals is smaller, and stabilises at 30.5. Withdrawals’ reported work ability at survey is 2.9 for those who respond 3–7 years after HDT-ASCT, and 3 for those who respond 8–12 years later. However, for those who respond more than 12 years later, it is 5.1. Work ability for non-withdrawals drops steadily to 7.5, 7.1 and 7. The trajectory for work ability in the withdrawal group suggests a rebound effect over time.

### Different trajectories: testing differences in means

We amplify the differences in trajectories for withdrawals and non-withdrawals in the last panel in Fig. 2. At diagnosis, work hours and work ability in the withdrawal group were around 90 and 80% of non-withdrawals’, respectively, but, while withdrawals’ work hours drop to zero, work ability rebounds to a level similar to where it started 13 years or more after treatment (Fig. 2). The difference in average weekly work hours between withdrawals and non-withdrawals is − 5.4 h at diagnosis, compared to − 29.4 h 13 years or more after treatment (Pr(|T| > |t|) = 0.0001). However, there is no statistically significant difference in average work ability (2.6 points lower in the withdrawal group at diagnosis, and 4 points lower after 13 years (Pr(|T| > |t|) = 0.265)).

### Economic loss estimation

The withdrawal group works on average 26.6 h less per week than non-withdrawals at survey. Using wage statistics for 2012 (year of survey start) from Statistics Norway, by gender and level of education, we estimate an expected mean hourly wage of NOK 265.40. This gives us a yearly loss per person of NOK 331,555 (EUR 34,791), equivalent to 70% of the average yearly wage in Norway in 2012 of NOK 470,900 (Statistics Norway).

## Discussion

Our data suggest a strong correlation between withdrawing from the labour market during illness and future labour market prospects, even when controlling for an extensive set of health variables. This brings us back to our introductory hypothesis: Withdrawal from work life has a causal and negative effect on future work participation. If our hypothesis holds true, there should be little or no change in the coefficient for employment before treatment (i.e. the opposite of withdrawal) as we expand the regression model with sociodemographic and health-related variables, and it should stay significant. This is precisely what we find. Thus, staying employed throughout diagnosis and treatment implies a consistently higher probability of future employment, regardless of symptom burden.

Our results are similar to other researchers’ findings on absenteeism; (sickness) absence leads to more absence, while being present (at work) promotes work participation. Studies using Norwegian administrative data, covering the whole population, show that compulsory dialogue meetings for long-term sickleave absentees reduced absence duration considerably [5]. Graded (instead of fulltime) sickleave had a similar effect, leading to shorter absence and higher subsequent employment rates [4]. The policy implications could be measures that promote inclusion throughout periods of illness and treatment, such as activity requirements [4].

Few studies have explored work life issues in long-term lymphoma survivors. A Danish registry-based study reported an increased risk of disability pension among survivors of haematological malignancies (including lymphoma) compared to the reference cohort [13]. In the patient cohort, comorbidity and need of treatment with anxiolytics and antidepressants after diagnosis were associated with disability pension. This is comparable with our findings of significant association with anxiety and employment, and in line with a previous study by our group among young adult cancer survivors of different diagnoses, where late adverse effects and other health-related factors were negatively associated with work life issues [9]. A qualitative study among self-employed cancer survivors, also found that late effects limited their work ability following treatment [14]. These findings emphasize the importance of addressing and initiating treatment and rehabilitation for late effects among cancer survivors in order to maintain work ability and function. A recent review concluded that multidisciplinary outpatient cancer rehabilitation might affect cancer patients’ physical and psychosocial status. However, they pointed to the need for more research on long-term outcomes, such as effects on return to work [15].

These findings implicate a need for both health care workers and those working in the welfare system to be aware of late effects after cancer as factors associated with reduced work life participation. Interventions aiming at improving late effects after cancer are strongly needed, and might improve work participation in the long term.

Work hours and work ability decrease for both withdrawals and non-withdrawals from diagnosis, through treatment, until survey when the participants receive the questionnaire. However, whereas both work ability and work hours amongst non-withdrawals stabilise relatively quickly at a modestly lower level, the trajectories are different for those who withdraw from the labour market. Their work hours drop below 10 in the first years after HDT-ASCT and continue to fall. A decade later, they hardly work at all. Work ability also drops but the initial fall is smaller, and the trajectory suggests a catch-up effect 13 years or more after treatment. The question arises: Is the difference in work ability between these two groups 13 years after HDT-ASCT identical to the difference that already existed at the time of diagnosis? And can the same be said for work hours? Does their withdrawal from work life stem not from lack of *ability*, but from lack of *possibility*?

Results from the standard t-test allow us to reject the null hypothesis for work hours, but not for work ability. We cannot conclude, on any conventional level of significance, that the difference in work ability 13 or more years after treatment is different from the difference in means that already existed at diagnosis, whereas for work hours it is highly significant. The ratios in Fig. 2 (last panel) illustrate this; while work ability for the withdrawal group starts just above 80%, drops to 40%, and rebounds to a level slightly below 80% of non-withdrawals’ work ability, their work hours drop from 90 to 0 % of non-withdrawals’.

If anything, we expect work ability to be under-reported for respondents outside the labour force, due to self-justification bias, i.e. they under-report their work ability to justify their exclusion [16]. Thus, we consider the gap between work ability and employment a lower bound.

Work hours in the withdrawal group does not reflect the catch-up effect in work ability. Assuming we could avoid their withdrawal, what is the potential gain from re-entering these patients into the labour force? Our estimations suggest a yearly loss of NOK 331,555 (EUR 34,791), equivalent to 70% of the average yearly wage in Norway in 2012 (Statistics Norway). Avoiding withdrawal and its subsequent effects could represent a substantial gain, not only for those directly affected but also for society as a whole.

### Norwegian labour market characteristics

Compared to other European countries, Norway has a relatively high employment rate. On the other hand, a relatively high share of the working-age population receives health-related benefits [17]. Work force participation depends on individual characteristics, such as education and work ability, but also on labour market regulations and the income insurance system.

### Strengths and limitations

Strengths of this study include the completeness and representativeness of the study population, with all lymphoma survivors after HDT-ASCT in Norway being accounted for, together with the high response rate of 77%. Furthermore, participants and non-participants are highly comparable, strengthening the generalisability of our results.

Our study is limited by the cross-sectional design preventing any conclusion regarding causality to be made. All work-related data were collected by questionnaire at one time point, with a risk for recall bias, and not controlled by interviews and/or data from The Norwegian Work and Welfare Administration.

## Conclusion

In this national study, we find that Norwegian lymphoma survivors have a higher probability of employment after treatment with HDT-ASCT if staying employed throughout diagnose and treatment. Our results support the hypothesis that withdrawal from the labour market has a negative effect on future labour market participation, even as we control for an extensive set of health variables. Thus, for a given symptom burden, withdrawal negatively affects employment later in life.

## Data Availability

The datasets used and/or analysed during the current study are available from the corresponding author on reasonable request.

## Abbreviations

BEAM: Carmustine, etoposide, cytarabine and melphalan
BMI: Body Mass Index
EUR: Euros
HADS: Hospital Anxiety and Depression Scale
HDT-ASCT: High-dose chemotherapy with autologous stem-cell transplantation
HL: Hodgkin lymphoma
NHL: Non-Hodgkin lymphoma
NOK: Norwegian kroner
OUH: Oslo University Hospital
REC South East: Regional Committee for Medical and Health Research Ethics of South-East Norway

## References

[1] Majhail NS, Ness KK, Burns LJ, Sun CL, Carter A, Francisco L (2007). Late effects in survivors of Hodgkin and non-Hodgkin lymphoma treated with autologous hematopoietic cell transplantation: a report from the bone marrow transplant survivor study. Biol Blood Marrow Transplant.

[2] Smeland K, Kiserud C, Lauritzsen G, Blystad A, Fagerli U, Fluge Ø (2013). High-dose therapy with autologous stem cell support for lymphoma in Norway 1987-2008. Tidsskr Nor Laegeforen.

[3] Kiserud C, Fagerli U, Smeland K, Fluge Ø, Bersvendsen H, Kvaløy S (2016). Pattern of employment and associated factors in long-term lymphoma survivors 10 years after high-dose chemotherapy with autologous stem cell transplantation. Acta Oncol.

[4] Markussen S, Mykletun A, Røed K (2012). The case for presenteeism—evidence from Norway's sickness insurance program. J Public Econ.

[5] Markussen S, Røed K, Schreiner RC (2017). Can compulsory dialogues nudge sick-listed workers back to work?. Econ J.

[6] Smeland KB, Loge JH, Aass HC, Aspelin T, Bersvendsen H, Bolstad N (2019). Chronic fatigue is highly prevalent in survivors of autologous stem cell transplantation and associated with IL-6, neuroticism, cardiorespiratory fitness, and obesity. Bone Marrow Transplant.

[7] Murbraech K, Smeland KB, Holte H, Loge JH, Lund MB, Wethal T (2015). Heart failure and asymptomatic left ventricular systolic dysfunction in lymphoma survivors treated with autologous stem-cell transplantation: a national cross-sectional study. J Clin Oncol.

[8] Tuomi K, Ilmarinen J, Jahkola A, Katajarinne L, Tulkki A (1998). Work ability index: Finnish Institute of Occupational Health Helsinki.

[9] Dahl AA, Fosså SD, Lie HC, Loge JH, Reinertsen KV, Ruud E (2019). Employment status and work ability in long-term young adult cancer survivors. J Adolesc Young Adult Oncol.

[10] Chalder T, Berelowitz G, Pawlikowska T, Watts L, Wessely S, Wright D (1993). Development of a fatigue scale. J Psychosom Res.

[11] Zigmond AS, Snaith RP (1983). The hospital anxiety and depression scale. Acta Psychiatr Scand.

[12] Vittinghoff E, McCulloch CE (2007). Relaxing the rule of ten events per variable in logistic and cox regression. Am J Epidemiol.

[13] Horsboel TA, Nielsen CV, Andersen NT, Nielsen B, de Thurah AJAO (2014). Risk of disability pension for patients diagnosed with haematological malignancies: a register-based cohort study. Acta Oncol.

[14] Torp S, Brusletto B, Withbro TB, Nygaard B, LJJoOR S (2020). Work experiences during and after treatment among self-employed people with cancer. J Occup Rehabil.

[15] Kudre D, Chen Z, Richard A, Cabaset S, Dehler A, Schmid M (2020). Multidisciplinary outpatient Cancer rehabilitation can improve Cancer patients’ physical and psychosocial status—a systematic review. Curr Oncol Rep.

[16] Black N, Johnston DW, AJJohe S (2017). Justification bias in self-reported disability: New evidence from panel data. J Health Econ.

[17] Øien-Ødegaard C, Reneflot A, Hauge LJ (2019). Use of primary healthcare services prior to suicide in Norway: a descriptive comparison of immigrants and the majority population. BMC Health Serv Res.

